# Ultra-High-Temperature Ceramic-Doped Inorganic Polymers for Thermo-Structural Fiber-Reinforced Composites

**DOI:** 10.3390/ma16206649

**Published:** 2023-10-11

**Authors:** Valentina Medri, Annalisa Natali Murri, Elettra Papa, Claudio Mingazzini, Matteo Scafè, Elena Landi

**Affiliations:** 1 National Research Council, Institute of Science, Technology and Sustainability for Ceramics (CNR-ISSMC), Via Granarolo 64, 48018 Faenza, Italy; valentina.medri@issmc.cnr.it (V.M.); elettra.papa@issmc.cnr.it (E.P.); elena.landi@issmc.cnr.it (E.L.); 2 SSPT-PROMAS-TEMAF, ENEA, Via Ravegnana 186, SP302, 48018 Faenza, Italy; claudio.mingazzini@enea.it (C.M.); matteo.scafe@enea.it (M.S.)

**Keywords:** fire resistant, fiber-reinforced composites, ultra-refractory ceramics, inorganic polymers, thermo-structural materials

## Abstract

New inorganic nanostructured matrices for fiber-reinforced composites with enhanced high-temperature stability were developed from alkali aluminosilicate polymers doped with different ultra-high-temperature ceramic (UHTC) particles. The alkali aluminosilicate matrices were synthesized at room temperature with a high SiO_2_:Al_2_O_3_ ratio and then further functionalized by doping with 4–5 wt % of micrometric SiC, ZrB_2_, ZrC, and HfC powders and finally thermally stabilized as glass–ceramics at 750 °C. The different UHTC-doped matrices were characterized according to their dimensional and microstructural changes after thermal cycling in air flux at 1000 °C. The first results showed that carbide-based UHTC powders improved the thermal stability of the matrices, preventing the excessive swelling of the material and the formation of detrimental voids that might result in the lack of adhesion with reinforcing fibers. Contrarily, the addition of ZrB_2_ resulted in an excessive matrix swelling at high temperature, thus proving no efficacy compared to the undoped matrix. Impregnation tests carried out on C-fiber fabrics showed good processability, adhesion to the fibers, and fracture pull-out, especially for carbide-based matrices.

## 1. Introduction

High-temperature-resistant matrices for fiber-reinforced composites have long been a well-established field of research due to the increasing demand for composites capable of combining excellent thermomechanical performance with simple, fast, and inexpensive processes. This is particularly true in the automotive and aeronautical sectors, where material solutions that exhibit good dimensional stability, excellent resistance to medium-high temperatures, and great versatility in production (similar to traditional Fiber-Reinforced Polymer (FRP) composites) are continuously sought [1,2].

In recent decades, traditional FRPs have been widely used as structural materials in various industrial fields, especially where it is necessary to minimize the mass of the system while ensuring the best structural response. This is thanks to their high specific strength, ease of manufacturability, low weight, and good chemical resistance [3,4,5,6]. Epoxy resins are commonly used as matrices for traditional FRPs, although there are several drawbacks that limit their wider use, such as their poor durability in humid environments, susceptibility to UV radiations, emissions of volatile organic compounds (VOCs), and most importantly, poor resistance to high temperatures and direct flames [7,8,9,10].

In addition to improving design flexibility and processing parameters (for example, through 3D-printing techniques [11]), current research efforts in the FRP sector are increasingly focused on enhancing the thermal resistance of organic resins to expand the range of applications, including those requiring operating temperatures above 400–500 °C [12,13,14,15,16,17,18].

In some cases, organic matrix composites can be replaced with ceramic or glass–ceramic matrix composites (CMCs), which have a remarkable resistance to high temperatures up to 1200 °C. However, their use is less versatile and their production process is more complex, energy-consuming, and expensive, and they cannot be designed for large-scale production of complex-shaped components [19,20]. Moreover, most CMCs have the disadvantage of showing poor resistance to oxidation. If exposed to highly oxidizing environments at high temperatures, they are prone to reacting with oxygen, resulting in material loss and a significantly lower protective effect [21,22,23,24].

Starting from these premises, in recent years safer and higher-performing matrices have been sought both from the point of view of thermal and fire protection and against detrimental oxidative processes without losing sight of the ease and cost-effectiveness of the process. Both scientific and industrial interests have been driven toward inorganic polymer-based composites, a subcategory of ceramic matrix composites (CMCs) which boast several attractive properties, including excellent high-temperature and fire resistance, good mechanical properties, and an outstanding tolerance to oxidation, corrosion, and aggressive environments [25,26,27,28,29].

Inorganic polymers with an alkali-aluminosilicate composition, such as geopolymers, are generally synthesized through the activation of an aluminosilicate solid precursor with alkali metal hydroxides, silicates, or phosphates at ambient temperature or slightly above and are characterized by good thermal and acid resistance, a low environmental impact, and a low cost [30,31]. The synthesis proceeds at a low temperature (generally a max of 80 °C) from the chemical activation of a wide range of precursor materials, such as natural or synthetic clays or even industrial wastes or mixtures of these materials, therefore also limiting the environmental impact and the energy demand of the whole manufacturing process [31,32]. Given their ease of processability and the possibility of working with the same equipment and within the same process conditions of traditional FRPs, inorganic polymers have been proposed as matrices for fiber-reinforced composites with the aim to substitute traditional FRPs where the working conditions require resistance to higher temperatures (400–500 °C) [33,34,35,36,37]. Lyon et al. [38] investigated for the first time the use of inorganic polymers with a poly(sialate-multisiloxo) structure, namely with a Si:Al ratio in the range of 18 to 35, as matrices for carbon-fiber-reinforced composites to be used as a safer alternative to composites based on flammable organic resins for aeronautic applications. The composite material they developed indeed did not ignite, reach flashover, or generate any smoke in a compartment fire test. Other studies investigated the fire protection ability of geopolymer and inorganic polymer-woven carbon and glass fibers [39,40], demonstrating their ability to meet fire requirements for aerospace sandwich structures and to retain most of their mechanical performances after fire exposure.

However, in most cases, the use of inorganic polymers as matrices for reinforcing fibers is precluded for applications at temperatures above 500–600 °C due to the physical transformations of the inorganic polymer. These transformations lead to the formation of a glass–ceramic material via densification, which results in swelling of the silicate phases of the system. Although the formation of such a silicate glass–ceramic surface can still have a beneficial effect on the matrix and protect the underlying fibers from oxidation by promoting self-healing mechanisms [41], the highly silicate nature of some inorganic polymers might lead to the swelling of the matrix itself in highly oxidizing environments. Such swelling behavior, in addition to negatively affecting the fiber–matrix adhesion and therefore the mechanical properties of the composite, can generate surface porosity and preferential oxygen entry channels, which can quickly lead to the degradation of the underlying fibers [35,36,42]. To increase the maximum working temperature and the overall heat resistance of inorganic polymers up to 1000 °C, compositions with lower Si:Al ratios, namely poly(sialate-siloxo), were investigated by He et al. [34]. The authors highlighted the better performances of these systems at high temperatures owing to the formation of highly stable mineral phases deriving from the crystallization of the inorganic polymer such as leucite and kalsilite. Nevertheless, they also disclosed a detrimental effect of such phase transformation; that is, a significant increase in the material’s porosity and crack formation after a remarkable volume shrinkage.

In this work, an innovative inorganic polymer matrix was designed from a commercial kaolinitic precursor and amorphous silica with the aim of ensuring an optimal balance between performance and cost. Such an inorganic polymer matrix was designed to enhance the thermo-structural properties of a fiber-reinforced composite, protecting the fibers even in highly oxidizing environments at high temperatures. This protection was achieved thanks to the formation of a stable amorphous glass–ceramic material through the vitrification of a doped high-silica polysialate resin [33,34,37,42,43,44,45]. Specific refractory fillers, such as silicon carbide (SiC), and ultra-high-temperature ceramics (UHTCs), namely zirconium diboride (ZrB_2_), zirconium carbide (ZrC), and hafnium carbide (HfC), were introduced into the matrix formulation to increase the thermal resistance of the composites due to their high melting point above 3000 °C [46,47,48,49,50,51,52]. The addition of refractory phases aimed to improve the physical behavior of the resin under high-temperature exposure by limiting its excessive swelling and avoiding damages to the protective outer surfaces, thus possibly improving the holding time of the composites in service. Technologically, the matrices were designed with a SiO_2_:Al_2_O_3_ molar ratio over 40 (i.e., a Si/Al molar ratio over 20) [38,39,43,44] to obtain the most suitable properties in the fresh state for properly impregnating the reinforcing fiber bundles.

Therefore, it is possible to obtain a thermo-structural material capable of working at service temperatures above 600 °C, pushing the limits of traditional polymer-based FRPs. Although not perfectly competitive with ceramic matrix composites (CMCs) in terms thermal properties, this new material can certainly boast enormous advantages in terms of production costs and process easiness. The use of a water-based inorganic resin, which can be processed in the same way as an organic resin, avoids any technological complications in the whole process. Moreover, more than 90% of the weight of the inorganic matrix is composed of extremely widespread and low-cost raw materials such as metakaolinitc clay and potassium silicate, while the ultra-refractory phases, with their relatively higher cost, affected only 5% by weight of the total starting components.

## 2. Materials and Methods

The inorganic polymer matrix formulations were prepared using a commercial metakaolin (MK) powder (Argical M-1200S from Imerys, France, D_50_ = 2 µm) and a potassium polysilicate aqueous solution (KSil) with molar ratios SiO_2_:K_2_O = 3.1 and H_2_O/K_2_O = 28 and a solid concentration of 36.0 wt %. Fused silica powder was used to set the total SiO_2_:Al_2_O_3_ molar ratio of the matrices to 40 (namely, a Si/Al molar ratio equal to 20) to provide polymeric characteristics to the materials by synthesizing a two-dimensional cross-linked poly(sialate-multisiloxo) [38,39,43,44]. Such a molecular structure characterized by a 2D cross-linked structure rather than a 3D network was specifically chosen because it has proven to have good mechanical properties and thermal resistance up to medium-high temperatures while at the same time exhibiting the ideal viscosity to properly impregnate a fiber reinforcement through vacuum-assisted techniques [38,39,43,44]. A reference formulation was therefore prepared with a plain, undoped inorganic polymer by placing the metakaolin and fused silica powders in a Teflon jar and then adding the liquid silicate solution and placing the closed jar in a planetary centrifugal mixer (Thinky Mixer ARE-500, Thinky Corporation, Tokyo, Japan). The jar was mixed for 5 min at 900 rpm and defoamed for a further 3 min at 900 rpm to avoid the formation of detrimental air bubbles. The reference formulation (M0) therefore contained 4.2 wt % metakaolin, 78.2 wt % potassium silicate, and 17.6 wt % fused silica, and its composition was defined using the following total molar ratios: SiO_2_:Al_2_O_3_ = 40, SiO_2_:K_2_O = 6.4, and Al_2_O_3_:K_2_O = 0.16. The true density of M0 after curing was 2.15 g/cm^3^ as determined by a He pycnometer.

Refractory powders were then used to functionalize the developed inorganic polymer and added to the basic M0 formulation. With the specific aim to further increase the high-temperature resistance of the inorganic polymer matrix and impart barrier properties against oxidative phenomena, different refractory powders were chosen as functionalizing fillers for the reference matrix M0.

The identified refractory powders were a silicon carbide β-SiC powder, grade BF 12 (H.C. Starck, Goslar, Germany); a zirconium diboride ZrB_2_, grade B (H.C. Starck, Germany); a zirconium carbide ZrC, 99.5% (Alfa Aesar, Ward Hill, MA, USA); and a hafnium carbide HfC, 99.5% (Cerac Inc., Milwaukee, WI, USA). Zirconium and hafnium carbide powders were specifically chosen for their inertness in the polysialate system even at high temperatures and for their extremely high melting temperature (3530 °C and 3900 °C, respectively) so as to provide increased thermal and dimensional stability to the materials. SiC and ZrB**_2_** powders were also chosen because of their ability to withstand high working temperatures and their good thermal shock resistance together with their ability to promote the formation of glassy phases at high temperature (SiO_2_ and ZrSiO_4_, respectively), which are thought to provide further protection against oxidative phenomena to a fibrous reinforcement [53,54,55]. The average dimensions of the powders (D10, D50, and D90) and values for the specific surface area (SSA) are listed in Table 1. Values were obtained from the technical data sheets provided by the producers or, where not available, via direct characterization using BET analysis in He-N2 flux (as for their specific surface area) (Sorpty 1750 BET, Carlo Erba Strumenti, Milan, Italy) and via X-ray particle size analysis (Sedigraph ET5100, Micromeritics, Norcross, GA, USA) (as for the particle size distribution).

Since the characteristics of the UHTC powders were very different, the criteria adopted in designing the doped formulations were the surface areas of the UHTC powders in contact with the slurry. Considering a surface area of 1 m^2^, for the sake of simplicity, the addition of UHTC powders was standardized to 1% by weight. The total amount of refractory powders was fixed at 5 wt % for all formulations calculated over the total weight of the final mixture (Table 2). After adding the refractory powders into the mixing jars, the slurries were further mixed in the planetary centrifugal mixer for 1 min at 900 rpm.

The resulting slurries were poured into plastic trays with dimensions of 35 mm × 35 mm and a thickness of 5 mm. The trays were then placed in an oven and cured at 80 °C for 24 h. After curing, the specimens were demolded and dried for an additional 12 h at 80 °C. Next, the specimens underwent a post-curing process via thermogravimetry for 1 h at 750 °C with a heating rate of 10 °C/min in an Ar flux of 30 mL/min to finally stabilize the matrix.

To evaluate the effect of the inorganic polymers as matrices for carbon fibers in high-temperature fiber-reinforced-composite applications, the slurries were used to cast prismatic samples with dimensions of approximately 8 mm × 12 mm and a thickness of 5 mm in silicon molds. Carbon fiber bundles extracted from a commercial bidirectional simple warping fabric (GG 220 P, 3K, 220 g/m^2^, 0.22 mm thick, G. Angeloni, Quarto d’Altino, Italy) were embedded in the samples. The fiber bundles, which had an average diameter of 7.4 µm (determined via SEM image analysis using an ESEM-FEI Quanta 200, Thermofisher Scientific, Waltham, MA, USA) and a theoretical density of 1.75 g/cm^3^, were manually pulled out from the fabric by unthreading the bundles of the weft from the warp. First, a 2 mm layer of matrix was deposited in the mold and partially consolidated at room temperature for 30 min to increase the surface tension of the material and make it suitable to arrange the bundle of reinforcing fibers without it settling on the bottom of the mold. Then, a second layer of matrix was deposited to cover the carbon fibers until the desired final thickness of 5 mm was achieved. These specimens were then cured at 80 °C for 24 h, demolded, and dried for an additional 12 h at 80 °C. Finally, the specimens were subjected to a post-curing step via thermogravimetry for 1 h at 750 °C using a heating rate of 10 °C/min. The fiber-reinforced samples were labeled as follows: M0-Cf, MS-Cf, MSZB-Cf, MSZC-Cf, and MSHC-Cf, depending on the matrix used (Table 2).

To evaluate the ability of such inorganic polymer matrices to be processed with traditional lay-up and vacuum bagging techniques, laboratory-scale demonstrators were produced and observed via SEM analysis. In detail, 30 mm × 30 mm 3-ply laminates were produced by manually impregnating the fiber fabrics with a roller soaked in the slurry, overlaying a next layer of C-fiber fabric, and repeating the operation for a final thickness of three layers. The impregnated fabrics were placed on a rigid support and covered with a layer of nylon fabric (peel-ply) and a layer of microperforated plastic film. The coated laminates were then placed into a 0.1–0.2 mm thick plastic vacuum bag sealed with heat-resistant rubber adhesive tape and in which a valve was positioned to create the vacuum. A dry scroll pump was then plugged into the valve, ensuring an inlet catchpot to prevent the liquid phases from damaging the pump, which was then operated at room temperature and ambient pressure until the matrix was completely dehydrated (2 to 5 h). The laminates were then extracted from the vacuum bag and cured for an additional 12 h at 80 °C until reaching a final thickness of about 1 mm after curing. Finally, the cured laminates underwent the post-curing treatment up to 750 °C in a tubular furnace (HST 12/600 220, Carbolite Gero, Neuhausen, Germany) under an Ar flux with a heating ramp of 2 °C/min and a final dwell of one hour at 750 °C before cooling down to 500 °C at 2 °C/min.

### Characterization of Materials

An environmental scanning electron microscope (ESEM-FEI Quanta 200, Thermofisher Scientific, Waltham, MA, USA) was used to characterize the matrices’ microstructure and the fiber/matrix interfacial zone in the samples. All specimens were coated with a conductive 5 nm layer of gold before being analyzed.

A simultaneous thermal analyzer (STA 449 C/4/G Jupiter, Netzsch Geraetebau Gmbh, Germany) was used in thermogravimetric mode to simulate the pyrolysis post-curing step of the composite specimens and to carry out the oxidation tests on the previously post-cured samples via a heating ramp of 10 °C/min up to 750 °C in an Ar flux and a 1 h holding time, a controlled cooling down to 30 °C (holding time: 30 min), and a second heating ramp up to 1000 °C in an air flux (30 mL/min) at 10 °C/min. An alumina support plate and zirconia protections were used at the base of the sample to prevent the samples sticking to the measuring plate.

X-ray diffractometric analyses (Bruker D8 Advance, Bruker—Karlsruhe, Germany) were carried out on the top surface of the composite samples in their as-given, post-cured, and oxidized states to investigate the phase changes in the matrix material upon thermal treatments.

Qualitative investigations of the microstructure at the fiber/matrix interface such as pull-out or debonding of fibers along the fracture surface of the composites were carried out by breaking the samples into two halves. For the sole purpose of evaluating the internal microstructure, the composite laminates were clamped with a mechanical vice onto a rigid support, keeping one half still while leaving the other half cantilevered. The cantilevered half was then bent by delivering a sharp blow to generate a fracture line. Subsequently, the two halves of each specimen were completely separated by repeatedly bending the sample along the identified fracture line. Such an out-of-standard procedure, which avoided ex post machining of the specimens, prevented the materials from delaminating and therefore from providing results distorted by possible stress at the interface due to mechanical cutting processes.

Tensile strength tests were finally performed on ad hoc molded and pyrolyzed laminated bars of standard dimensions (250 × 25 × 3 mm) using a Zwick-Roell Z050 universal testing machine (Ulm, Germany) in displacement control mode.

## 3. Results and Discussion

### 3.1. Microstructural Evaluations

**Figure 1:**
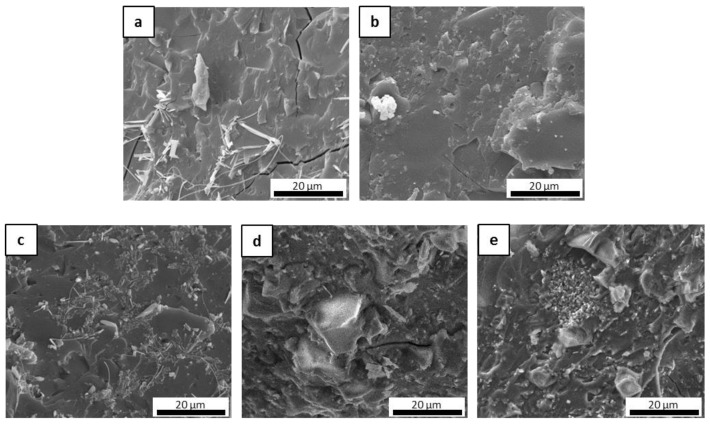
Microstructures of the as-given cured matrices: M0 (**a**); GS (**b**); MSZB (**c**); MSZC (**d**); MSHC (**e**).

The microstructure of the fracture surfaces of the inorganic polymer matrices after the curing step at 80 °C was investigated via scanning electron microscopy (SEM). All materials were well consolidated (Figure 1). Superficial fissures were observed only in M0 (Figure 1a), which resulted in it being more prone to cracking in comparison to the other matrices due to the absence of the ceramic fillers. In general, the fillers hindered the propagation of dehydrating cracks through the matrix [34] and improved the toughness of the material, contributing to the formation of a more cohesive network that could limit the triggering of fractures. This was evident when observing the other samples, in which the ceramic fillers appeared to be well distributed and incorporated within the geopolymer phase as based on their granulometry (Figure 1b–e). The selected powders differed in their physical properties in some respects, especially in regard to their specific surface area, which ranged from ~0.9 to 11.6 m^2^/g for ZrC and SiC, respectively. Characteristic diameters varied accordingly, with D_50_ ranging from around 1 µm for SiC and HfC up to 7.3 µm for ZrC. The HfC powder had smaller dimensions than ZrC, which contributed to forming a more uniform microstructure in the MSHC sample compared to the MSZC sample. Newly formed acicular crystal structures were randomly observed in M0 (Figure 1a) and were consistent with potassium bicarbonate salts that formed after the samples were exposed to the environmental atmosphere during storage.

### 3.2. Thermal Behavior

**Figure 2:**
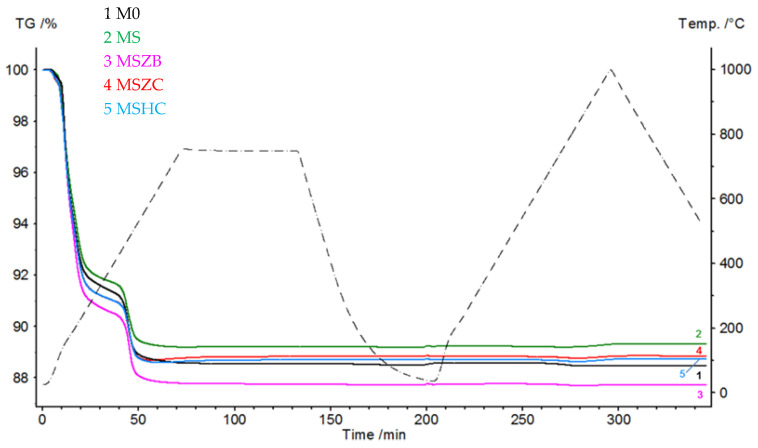
Thermogravimetric curves of matrix samples subjected to a cycle of pyrolysis at 750 °C and oxidation at 1000 °C (Ar at 30 mL/min, 30–750–30 °C, and 10 °C/min; air at 30 mL/min, 30–1000–500 °C, and 10 °C/min).

Figure 2 shows the thermogravimetric curves (TGs) of the matrices that were subjected to a pyrolysis cycle in Ar and oxidation at 1000 °C in an air flow. During the pyrolysis step, the curves had similar trends, with the most significant mass loss concentrated in the 120–220 °C range, which was due to the evaporation of the adsorbed water (6.6–8.4% for MSZC and MSZB, respectively). The second mass loss event, which was due to the dehydroxylation of the silicate phases, was recorded in the range of 440–520 °C for all samples, equal to approximately 2% by mass for all formulations. Finally, beyond 600 °C, a slight increase in mass was noted in the MSZC and MSHC samples; this was attributed to the slight oxidation of carbides caused by the oxygen-bearing matrix.

**Figure 3:**
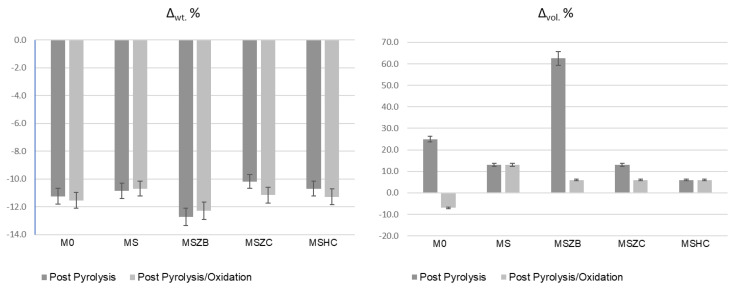
Weight loss and dimensional changes in inorganic polymer matrices after pyrolysis and pyrolysis/oxidation cycles (error = 5%).

**Figure 4:**
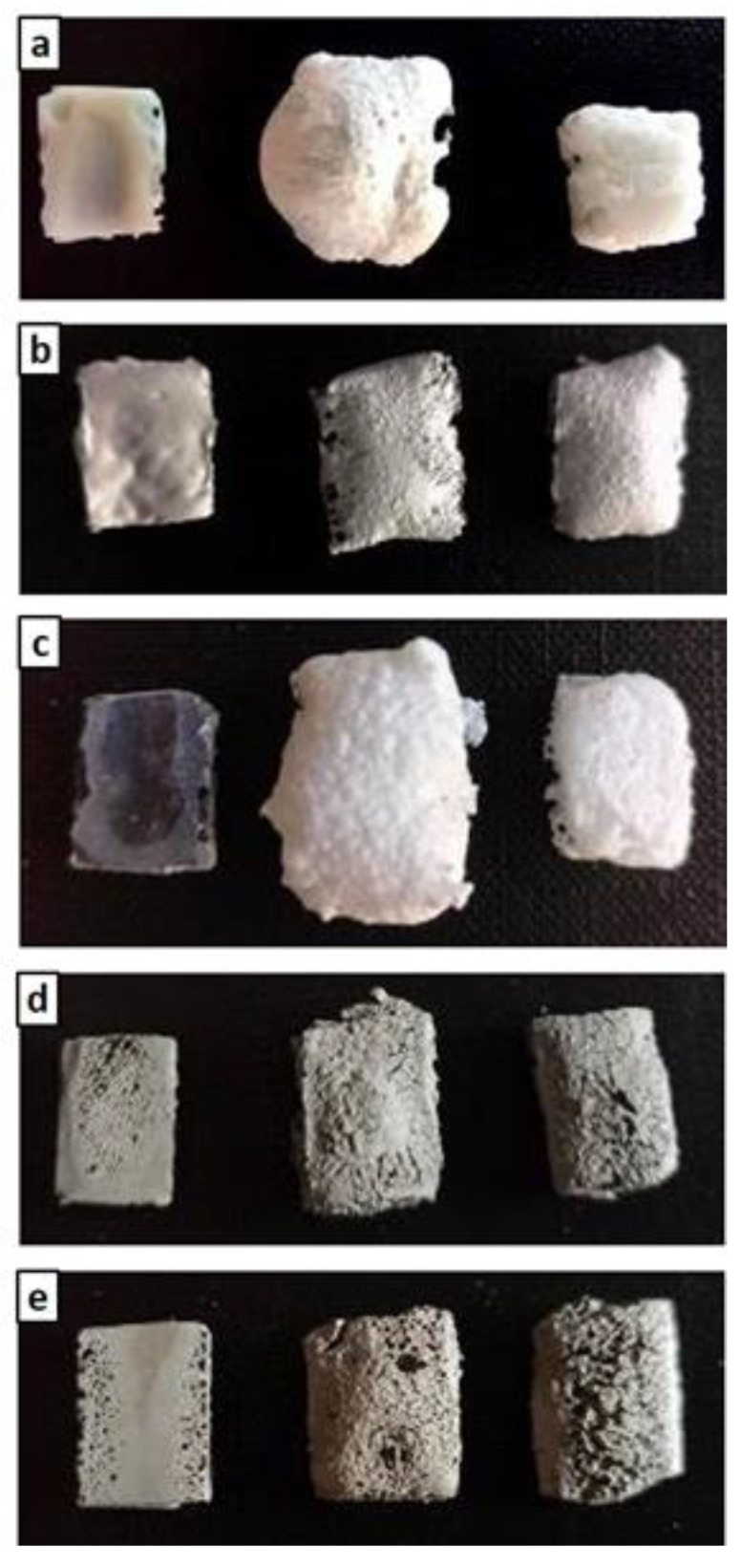
As-given (left), after post-curing (center), and after post-curing/oxidation cycle (right) for M0 (**a**), MS (**b**), MSZB (**c**), MSZC (**d**), and MSHC (**e**).

In the oxidation step, it can be observed that the materials were stable during the thermal cycle of exposure at 1000 °C, as confirmed by the straight line of the curves for all materials from the beginning of the second heating phase to the final temperature. There was a slight inflection of the curves in the temperature range around 800 °C, with a slight increase in mass (~0.1%) following a similar loss in weight. Such a slight increase in mass, presumably due to SiC oxidation of the matrices, was not visible in the sample formulated with amorphous silica alone (M0) or in MSZB, where the formation of gaseous B_2_O_3_ might have compensated for the mass gain due to the oxidation of SiC and ZrB_2_ to ZrO_2_ as confirmed by the XRD analysis (this will be discussed further later). The post-curing cycle generated marked swelling in all tested samples (Figure 3 and Figure 4) due to the evolution of gas from the highly silicate matrix owing to the evaporation of the adsorbed water. Consistent with the trend reported in the TG curves shown in Figure 2, such swelling was more significant in the M0 sample, which did not contain any UHTC ceramic phases, and in MSZB. In samples containing only carbide phases, the phenomenon was observed in a limited way.

The high swelling observed in the MSZB sample was likely due to the retention of a higher amount of adsorbed water, which was favored by the presence of the highly hygroscopic B_2_O_3_ as a surface impurity on the ZrB_2_ particles [56,57] despite ZrB_2_ only constituting 0.5 vol % of the consolidated MSZB. The subsequent oxidation heat treatment at 1000 °C resulted in marked viscous flow with consistent sintering effects and dimensional shrinkage for the M0 and MSZB samples. Contrarily, the carbide-doped samples did not exhibit any significant volume change compared to the post-curing treatment. In particular, in the MSZC and MSHC samples, the heavy and almost inert particles, which were well distributed in the matrix, pinned and broke the forming bubbles, providing dimensional stability to the matrices. Overall, the limited swelling in the carbide-doped samples indicated a more marked refractoriness compared to the plain inorganic matrix and the boride-doped one.

**Figure 5:**
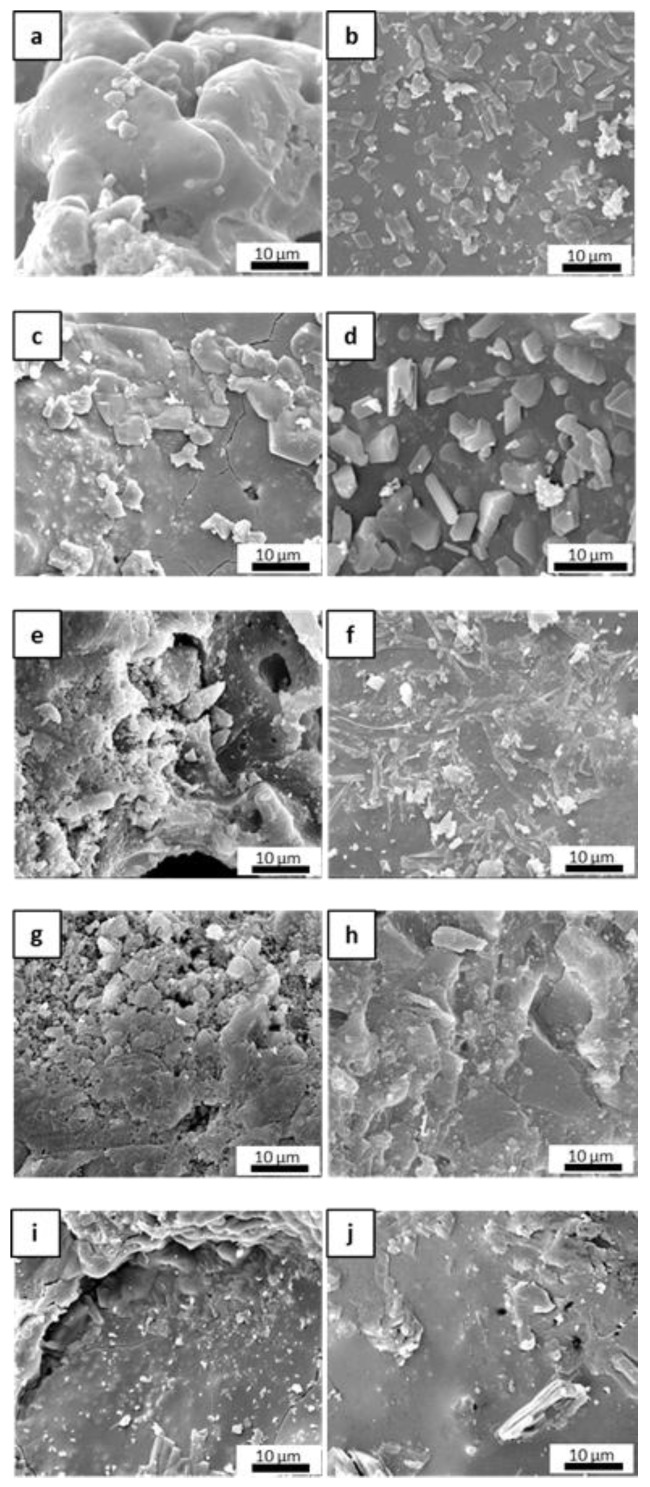
The inorganic polymer matrices M0 (**a**,**b**), MS (**c**,**d**), MSZB (**e**,**f**), MSZC (**g**,**h**), and MSHC (**i**,**j**) after pyrolysis (**a**,**c**,**e**,**g**,**i**) and after pyrolysis/oxidation cycle (**b**,**d**,**f**,**h**,**j**).

Figure 5 shows the microstructural evolution of the matrices after the post-curing and oxidation treatments. After post-curing, M0 exhibited a lower-order inner porosity in the glassy matrix, while MS and MSZB exhibited the formation of an amorphous superficial layer with newly formed crystals and cracks. In the MSZC and MSHC samples, the glassy phase was less prominent, with micrometric pores among the grains. The oxidation step at 1000 °C resulted in the expected further densification of the matrices via viscous flow at higher temperatures and a denser microstructure in all the samples.

### 3.3. Phase Composition

**Figure 6:**
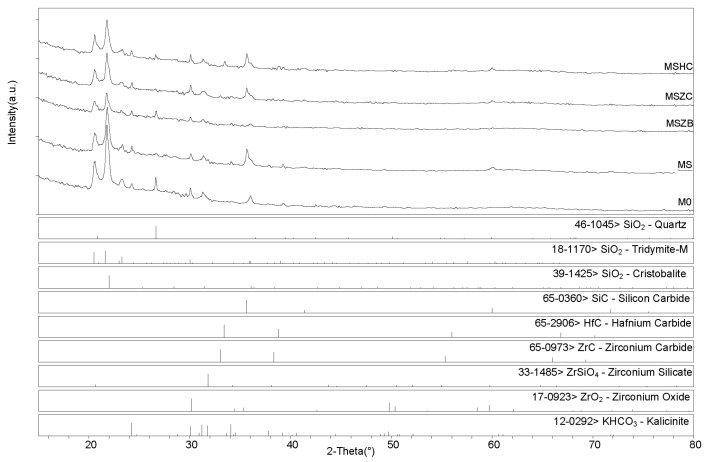
XRD patterns for samples after pyrolysis treatment.

**Figure 7:**
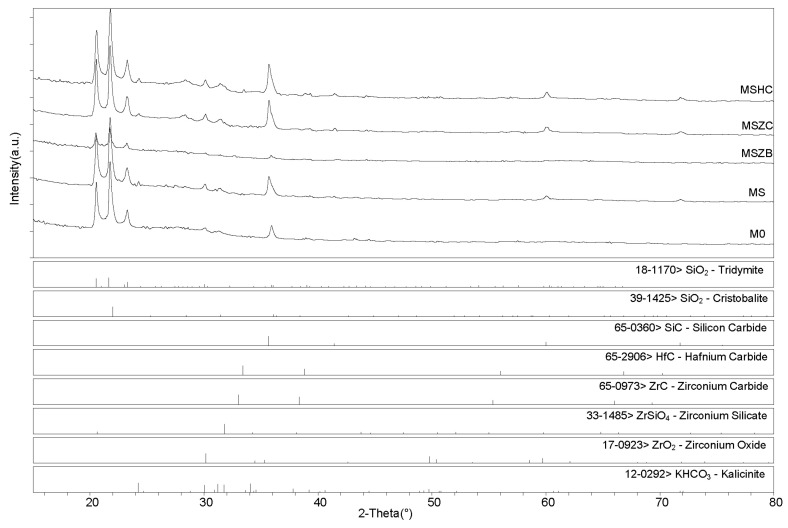
XRD patterns for samples after pyrolysis/oxidation treatment.

X-ray diffractometry (XRD) patterns collected for the post-cured samples showed a similar phase composition for all samples (Figure 6 and Figure 7), with the main identified crystalline phases being SiO_2_ polymorphs such as quartz, tridymite, and cristobalite and the latter being the two high-temperature forms generated upon heating at 750 °C. Secondary phases included SiC, which was detected in MS, MSZC, and MSHC, as well as ZrC and HfC, which were detected in trace amounts in MSZC and MSHC, consistent with the starting formulations. A slightly different phase composition was identified for MSZB, which did not show any presence of SiC, contrary to its starting formulation, and had significantly lower signals for crystalline SiO_2_ phases, resulting in a more amorphous pattern. In this sample, ZrB_2_ particles were likely oxidized despite the inert environment, forming ZrO_2_ (found in trace amounts) and liquid phases of B_2_O_3_, which were responsible for its final glassy surface after being treated to 1000 °C (Figure 4).

The presence of zirconium oxide and zirconium silicates in the spectrum indicated the instability of the ZrB_2_ UHTC phase and its reactivity with the matrix system at the considered temperature, which suggested limited effectiveness in improving the thermo-structural properties of the material. The absence of SiC was likely due to the highly reactivity of the system created by the decomposition of ZrB_2_, which promoted a highly oxidizing environment, in turn resulting in the oxidation of SiC. Although traces of SiOC were hardly detectable, they cannot be excluded due to peak overlapping with other more prominent phases (i.e., kalicinite). Additionally, as observed in microstructural examinations, potassium bicarbonate (KHCO_3_) was spotted in all samples; it derived from the post-storage environmental carbonation of the free alkali in the samples due to their reaction with H_2_O and CO_2_.

After post-curing/oxidation treatments, the samples maintained the same phase composition with no evidence of newly formed phases nor crystalline reaction products upon oxidation except for the absence of quartz from the SiO_2_ polymorphs. A higher degree of crystallinity was also evidenced by the sharper peaks defining the main detected phases. As previously reported for the post-curing treatment, the MSZB sample did not show the presence of SiC. All samples showed evidence of post-storage environmental carbonation as demonstrated by the detection of kalicinite traces.

### 3.4. Evaluation of the Fiber–Matrix Interface

**Figure 8:**
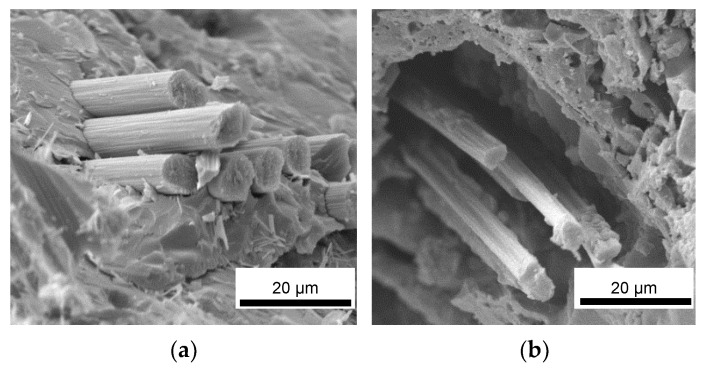
M0-Cf fiber-reinforced composite: as-given (**a**) and after post-curing (**b**).

To evaluate the protective effect of the inorganic matrix on carbon fibers, the cross sections of samples with embedded fibers (M0-Cf, MS-Cf, MSZB-Cf, MSZC-Cf, and MSHC-Cf) were examined via electron microscopy to investigate the interfacial zone and evaluate the occurrence of vitrification of the inorganic matrix on the fiber bundles after the post-curing and oxidation treatments. All the material cured at 80 °C exhibited a very good cohesive interfacial zone with fibers tightly attached to the surrounding matrix (Figure 8). At the macroscale, the post-curing treatment at 750 °C had a detrimental effect on fiber adhesion given the significant thickness of the matrix layers relative to the fibers. This was particularly evident for the highly swollen samples (M0-Cf and MSZB-Cf), in which gas evolution in the matrix promoted the formation of large cavities, which in turn partially left material voids around the fibers’ cross section (Figure 8b).

**Figure 9:**
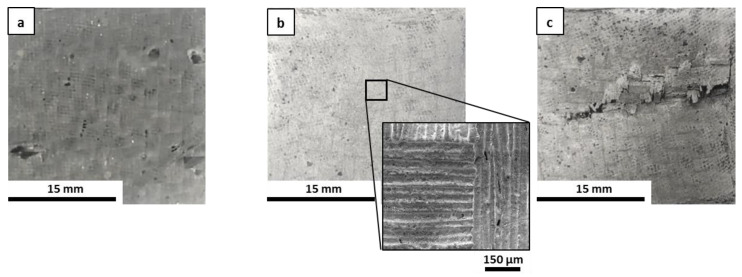
MSZC-Cf laminate before pyrolysis (**a**), after pyrolysis (**b**), and break-up (**c**).

However, the presence of the reinforcement and the local adhesion between the matrix and the C-fibers acted as a mechanical hindrance to the swelling of the silicate phases of the matrix, which was mainly observed on the top surface of the samples. This resulted in a denser and more compact microstructure around the reinforcement, thus maintaining the mechanical integrity of the composite. As previously mentioned, the carbide-doped matrices exhibited limited swelling after pyrolysis, mostly on the top surface of the samples. However, they maintained a remarkably tight adhesion to the reinforcing fibers in the denser middle of the cross section, thus protecting the fibers along their entire surfaces and likely avoiding any mechanical strength losses. Figure 9 shows images of the MSZC-Cf laminate (as an example of all samples) after the curing step at 80 °C, after post-curing, and after bending breakage. It is evident that the post-curing treatment did not result in any swelling of the matrix material—contrary to what was reported for the bulk matrix specimens—due to the limited amount of material deposited in the thin interlayers of the laminates and the beneficial effect of vacuum-assisted infiltration. The thermal process caused the partial vitrification of the matrix, locally sealing the small surface imperfections due to irregular impregnation, thus forming a more protective layer on the material [41,58].

**Figure 10:**
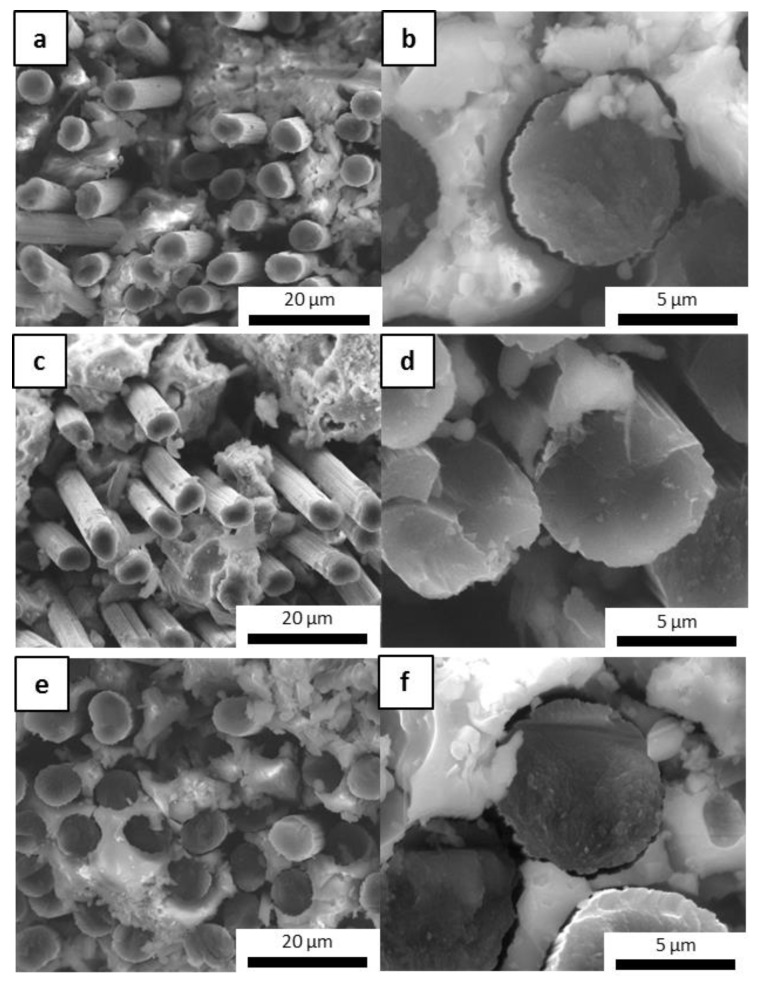
Fracture surface of pyrolyzed laminates MSZB-Cf (**a**,**b**), MSZC-Cf (**c**,**d**), and MSHC-Cf (**e**,**f**).

**Figure 11:**
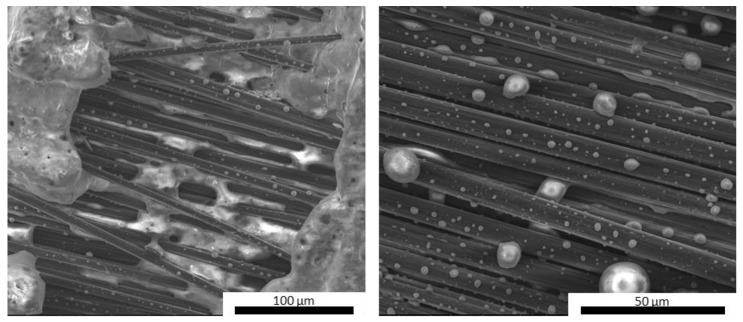
Upper surface of post-cured MSZB-Cf laminate.

Upon fracturing, it can be observed that the laminates exhibited the typical crack pattern of a tough material, where the proper adhesion between the fibers and the matrix promoted the triggering of pull-out phenomena and fracture energy dissipation. Indeed, upon observing the microstructure of the fracture surface of the laminates (Figure 10), it can be noticed that the fibers were properly embedded in the inorganic polymer, showing no detachments all along the fiber surface or detrimental chemical interactions at the fiber/matrix interface. This confirmed that a relatively low post-curing temperature of 750 °C can promote the partial densification of the inorganic polymer, which in turn locally tightens to the fibers, thus promoting a mechanically adequate adhesion. Overall, the presence of UHTC powder doping, given the micrometric particle size and even distribution in the materials, did not result in particle clusters among the fiber bundles, thus avoiding the formation of detrimental defects in the composites. However, unlike the carbide-doped matrices, the MSZB-Cf formulation proved to have a less protective effect on the reinforcing fibers and therefore on the whole composite. This was due to the significant amount of liquid phases formed upon the post-curing step by decomposition of ZrB_2_, which ultimately resulted in a marked differential shrinkage of the vitrified matrix on the laminate upper surface (Figure 11). The MSZB-Cf composite surface, which was unevenly coated and had localized voids of significant size, led to the formation of uncovered areas in which the fibers were completely exposed and easily subjected to progressively degrading oxidative phenomena, significantly limiting the overall performances of the material.

### 3.5. Mechanical Properties

Standard specimens for tensile strength tests were obtained by impregnating carbon fiber fabrics with the SiC-doped matrix formulation (MS) through hand lay-up and a vacuum bagging apparatus. Ten layers of fabrics were stacked with alternating orientations of 0–90° to obtain a final thickness of about 3.5 mm and then cured at 80 °C and post-cured at 750 °C under an Ar flux. The MS formulation was chosen as the representative for all formulations doped with 5 wt % carbides, as it has been previously demonstrated that such refractory fillers do not interact with the aluminosilicate environment and physically behave like inert fillers. The tensile strength and elastic modulus values were then determined on four samples according to ASTM D3039 [59]. Both ends of all the specimens were bonded with tapered tabs to avoid tension concentration with the shear load, providing a final available area of 80 ± 0.5 mm^2^.

All specimens showed valid failure modes according to ASTM D3039 (lateral gage top (LGT) or lateral gage bottom (LGB)) without any occurrence of failure due to a shear or delamination mode, suggesting a good adhesion between the fibers and the matrix. The obtained mean values for MS-Cf were 136.7 ± 3.4 MPa and 37.4 ± 2.9 GPa for the tensile strength and Young’s modulus, respectively, with an ultimate strain of 1.4 ± 0.1%.

The data obtained show good reproducibility even if the final strength values are slightly lower than those reported in the literature for similar composite materials. Hammell et al., reported mechanical properties of a bidirectional C-fiber-reinforced polysialate, showing ultimate tensile strength values of 332 MPa and an elastic modulus of 76 GPa [40], while Mills et al., reported a tensile strength of 288 MPa and an elastic modulus of 32 GPa for a SiC-fiber-reinforced polysialate of a commercial type (PyroSiC from Pyromeral, Barbery, France) [45] (Table 3).

The differences found in the values of the tensile strength and tensile elastic modulus certainly depend on the different formulation of the matrix developed in this study compared to the other sialate-based matrices, especially in terms of the Si/Al ratio. In particular, we noted that the values obtained experimentally from MS-Cf are lower than the previous references both in terms of stiffness and ultimate strength together with the relatively high failure deformation values, which are higher than those previously reported (0.67% [40] and 1.0% [45]). Most likely, the level of structural disorder and the presence of non-bridging oxygens tends to decrease the elastic modulus values for highly silicatic compositions, as in this case (Si/Al = 20) [60], resulting in higher ultimate strain and lower strength values. Not least, the lower tensile properties of the tested samples might be due to fiber misalignments and manufacturing defects likely derived from the manual sample processing, which caused a smaller volumetric fraction of fibers to be aligned with the loading direction, a factor which significantly influenced the final tensile response of the cross-ply laminates [61].

However, on an overall basis, the obtained results can be considered comparable with other high-temperature-resistant and fiber-reinforced composites, especially when taking into account the processing advantages that characterize their production processes. Table 3 reports literature data for other fiber-reinforced composite materials tested under a uniaxial tensile load in a cross-ply 0–90° configuration. CMCs based on non-oxide matrices can exhibit tensile properties at the same order of magnitude (also in the range of 200–250 MPa) depending on the processing conditions and the specific material combination [62,63,64,65], but they would be significantly more expensive and complex to produce. The same applies for oxide-based matrices [66] and glass–ceramic matrices [67,68,69], for which the complexity of the manufacturing process can affect costs, as chemical vapor infiltration (CVI), polymer infiltration and pyrolysis (PIP), or hot-pressing (HP) may be more expensive than other processing routes.

**Table 3:** Properties of cross-ply laminate composite materials with different fiber/matrix compositions. Legend: SI: slurry infiltration; DPY: densified preformed yarn; HI-VB: hand impregnation + vacuum bagging.

Matrix	Fibers	Process	Fiber Fraction	Tensile Strength	Ultimate Strain	Young’s Modulus	Reference
			(vol %)	(Mpa)	(%)	(Gpa)	
K-polysialate	SiC	HI-VB	30	288	1.0	32	[45]
K-poly(sialate siloxo)	C	HI-VB	50	343	-	79	[38]
K-polysialate	C	HI-VB	50	332	0.67	76	[40]
SiC	SiC	CVI	40	255	0.47	230	[62]
SiC	C	CVI	40	204	0.35	88	[63]
C	C	DPY	60	225	0.25	100	[64,65]
Mullite-SiOC	Al_2_O_3_	PIP	50	181	-	98	[66]
Al_2_O_3_	Al_2_O_3_	SI	37	170	-	145	[66]
LAS glass	SiC	HP	46	285	-	-	[65,67]
BMAS glass	SiC	HP	35	236	0.84	98	[68]
CAS glass	SiC	CVI	34	220	0.83	110	[65,69]
DGEBA epoxy + 5%SiC	C/Glass/Kev	HI-VB	60	322	-	14	[70]
PEK	C	HI-VB	-	425	9.4	7.8	[71]
DGEBA epoxy	C	HI-VB	-	311	11.3	5.2	[71]
Araldite epoxy	C	HI-VB	40	425	5.0	8.7	[72]
Araldite epoxy	Glass	HI-VB	40	112	4.0	2.9	[72]
MS0-Cf K-poly (sialate-multisiloxo)	C	HI-VB	35	136.7	1.4	37.4	-

It is clear that composite materials with a polysialate matrix such as the one investigated here cannot boast the same operating temperatures as traditional CMCs, since at temperatures above 1000 °C, they can be subject to instability phenomena (partial crystallization and consequent generation of stress at the fiber/matrix interface); however, when limited to temperatures in the same range as the process temperatures (700–750 °C), they can boast various advantages. Like most glass–ceramic matrices, sialate-based matrices can also provide the ability to vary the chemical composition depending on the performance required, allowing for more flexible designs to meet specific application requirements. In this case, the addition of refractory fillers allowed the maximum operating temperatures to be increased, keeping costs relatively low. Furthermore, the specific formulation of this matrix allowed the final weight of the components constructed to be kept low compared to other CMCs, resulting in the advantage of overall structural lightening. Furthermore, they have proven to be resistant to oxidation and to maintain their structural integrity even in oxygen-rich environments, making them possibly suitable for applications in combustion environments, rocket nozzles, and gas turbine components as well.

However, the most remarkable advantage provided by these materials and the related technology is certainly inherent to the process phase, which allows the use of water-based slurries (safe for human health and sustainable from an environmental point of view, eliminating the need for solvents or materials with high VOC emissions) and impregnation processes that are completely compatible with the instrumental technologies traditionally used for the production of PMCs [69,70,71]. This in turn would also facilitate the production of components without particular dimensional or geometric constraints, since the production of laminates in complex and large dimensions is exclusively limited by the dimensions of the ovens for the pyrolysis step. Finally, also in terms of costs, these materials present exceptional advantages compared to other CMCs, since the whole production process is significantly less energy- and time-consuming compared to, for example, CVI, PIP, or HP, and the raw materials used for the formulation of the slurries are certainly cheaper (the functionalizing refractory fillers, which have significantly higher costs than metakaolin and silicate, are introduced at a rate of only 5% and therefore do not affect significantly the final cost of the products).

## 4. Conclusions

Novel inorganic polymer-based matrices were successfully developed for the production of thermo-structural fiber-reinforced composites. These matrices were constructed from an alkali aluminosilicate glass–ceramic material with a significantly high SiO_2_:Al_2_O_3_ molar ratio of 40, which promoted the formation of a 2D polysialate network, in turn favoring the impregnation process of fiber fabrics (thanks to its low viscosity) and allowing the subsequent vitrification of the amorphous phases of the materials by post-curing treatments at medium-high temperature. Such an amorphous glass–ceramic material was able to seal the voids in the outermost layers of the composites and thus guaranteed the protection of the reinforcement from high temperature oxidative processes.

The addition of carbide-based UHTC phases (SiC, ZrC, or HfC) to the formulations in quantities of only 4–5 wt % increased the stability of the materials, reducing weight loss at high temperature, albeit to a limited extent. However, the addition of refractory ceramic fillers significantly improved the dimensional stability of the matrix, particularly during post-curing. These fillers prevented excessive swelling or shrinkage via viscous flow at high temperature, limiting the formation of voids and surface defects in the fiber-reinforced laminates. During post-curing, the carbide-doped materials experienced a limited dimensional increase of about +6–13%, whereas the undoped and boride-doped matrices exhibited swelling of 25% and over 60%, respectively. The addition of ZrB_2_ was found to be less effective than the undoped reference matrix due to the instability of ZrB_2_ and its tendency to form B_2_O_3_ at relatively low temperatures, with the consequent volatilization of gaseous components during high temperature cycling, significant material loss, and formation of porosity and surface voids on the composites. Again, the oxidation treatment at 1000 °C resulted in a remarkable dimensional shrinkage for the undoped and the boride-doped matrices due to the consistent sintering effect via the viscous flow of liquid phases, while the carbide-doped materials showed almost no dimensional changes compared to the post-curing step.

All formulations proved to be suitable for impregnating fiber fabrics thanks to the sufficiently low viscosity of the inorganic resins in the fresh state and to the micrometric dimensions of the UHTC fillers, which did not create obstructions between the bundles of fibers. Finally, vacuum bagging impregnation techniques for the production of laminates resulted in significantly thin matrix layers among the fiber fabrics, which mostly avoided the development of air bubbles and the formation of macrocavities inside the samples, as was the case for the massive bulk specimens.

These are the first results of promising research currently underway. Further studies will be conducted to optimize the curing and post-curing conditions on specimens of more significant dimensions, to validate the results on a larger scale, and to investigate the residual mechanical properties following heat treatment and direct flame. Also, the industrial ability of the process will be more thoroughly investigated; namely, the possibility of producing prepregs that can be stored for long periods without a loss in properties.

## Figures and Tables

**Table 1:** Powder characterizations (* data provided by the producer).

	Density (g/cm^3^)	D_10_ (µm)	D_50_ (µm)	D_90_ (µm)	SSA (m^2^/g)
β-SiC	3.21	0.30 *	1.10 *	2.07 *	11.6 *
ZrB_2_	6.10	1.15	2.92	4.70	1.0 *
ZrC	6.73	1.72	7.30	17.28	0.87
HfC	12.69	0.29	0.80	5.0	1.19

**Table 2:** Inorganic polymer formulations. The reference slurry (M0) was composed of 4.2 wt % metakaolin, 78.2 wt % potassium silicate, and 17.6 wt % fused silica.

	Composition (wt %)				
Sample	M0	SiC	ZrB_2_	ZrC	HfC
M0	100	0	0	0	0
MS	95	5	0	0	0
MSZB	95	4	1	0	0
MSZC	95	4	0	1	0
MSHC	95	4	0	0	1

## Data Availability

There is no supplementary material in the authors’ possession to be disclosed. All data presented in the manuscript are the property of the authors.
